# Occurrence of *Leishmania* spp. in Roadkilled Wild Mammals on Highways in the State of São Paulo, Brazil

**DOI:** 10.1590/0037-8682-0067-2024

**Published:** 2025-12-19

**Authors:** Silvia Juliana Ortiz Garavito, Islam Hussein Chouman, Pedro Enrique Navas Suarez, Maria Alejandra Arias Lugo, Ricardo Augusto Dias, José Luiz Catão Dias, Márcia Dalastra Laurenti, Vânia Lúcia Ribeiro da Matta, Claudia Momo

**Affiliations:** 1 Universidade de São Paulo, Faculdade de Medicina Veterinária e Zootecnia, Departamento de Patologia, São Paulo, SP, Brasil.; 2 Universidade de São Paulo, Faculdade de Medicina, Departamento de Patologia, Laboratório de Patologia de Moléstias Infecciosas (LIM-50), HCFMUSP, São Paulo, SP, Brasil.; 3 Centro Universitário das Américas (FAM), São Paulo, SP, Brasil.; 4 Universidade de São Paulo, Faculdade de Medicina Veterinária e Zootecnia, Departamento de Medicina Veterinária Preventiva e Saúde Animal, São Paulo, SP, Brasil.

**Keywords:** Roadkill, Passive surveillance, Leishmaniasis, Zoonoses, One health

## Abstract

**Background:**

Leishmaniasis undergoes geographic expansion in the Neotropical regions, driven by ecological and socioeconomic factors that contribute to urban and peri-urban outbreaks. Roadkilled wild mammals represent a potential source of eco-epidemiological data, and polymerase chain reaction (PCR)-based detection provides a sensitive tool for the surveillance of *Leishmania* spp.

**Methods:**

This study investigated the occurrence of *Leishmania* spp. in 40 roadkilled wild mammals collected along highways in the central-western and northern coastal regions of São Paulo State, Brazil between 2020 and 2022. Necropsies were performed on all specimens, and ear skin samples were collected for molecular detection of *Leishmania* spp. using primers targeting kinetoplast DNA (kDNA). Positive samples were further analyzed for species identification by PCR-restriction fragment length polymorphism (RFLP) targeting the *hsp70* gene. Restriction patterns were compared with reference strains to confirm *Leishmania* species identity.

**Results:**

*Leishmania* spp. kDNA was detected by molecular diagnosis in skin tissue samples from several roadkilled wild mammals collected in the state of São Paulo. Subsequent species-level identification using hsp70 PCR-RFLP detected *Leishmania infantum chagasi* in a maned wolf (*Chrysocyon brachyurus*) and *Leishmania amazonensis* in a raccoon (*Procyon cancrivorus*).

**Conclusions:**

Wild mammals could be exposed to distinct *Leishmania* species across diverse ecological contexts, and examining roadkilled animals, combined with the molecular detection of parasites, proved to be an effective tool for passive *Leishmania* surveillance, highlighting the need for integrated investigations within a One Health framework.

## INTRODUCTION

Leishmaniasis is a zoonotic disease caused by protozoa of the genus *Leishmania*, which are transmitted by phlebotomine sand flies and maintained in complex cycles involving domestic and wild mammals, including humans, dogs, rodents, and marsupials^1^ ^-^ ^4^. Their distribution is influenced by environmental, socioeconomic, and climatic factors, such as deforestation, reservoir diversity, and conditions favorable to vector survival^4^ ^-^ ^6^.

In Neotropical regions, American Visceral leishmaniasis (AVL) and American tegumentary leishmaniasis (ATL) differ substantially in clinical, epidemiological, and diagnostic aspects. In Brazil, AVL is a systemic disease caused by *Leishmania (L.) infantum* and transmitted mainly by *Lutzomyia longipalpis*, with domestic dogs being the primary urban and peri-urban reservoir^2^ ^,^ ^4^. ATL, mainly caused by *Leishmania (V.) braziliensis*, predominates in rural or sylvatic settings. It involves vectors, such as *Nyssomyia whitmani* and *Ny. Intermedia*, and is maintained primarily in reservoirs of wild mammals, such as rodents, marsupials and carnivores^2^ ^-^ ^4^. However, canine leishmaniasis ranges from asymptomatic to chronic multisystemic disease, which differs from human cases. Dogs may harbor *L. infantum* on intact skin without lesions, highlighting its epidemiological role in parasite transmission^7^ ^,^ ^8^.

In Brazil, ATL in the southeastern region were first reported in the late 19th century, with early cases associated with rural migration and road construction^9^ ^,^ ^10^. By contrast, AVL expanded west to east in São Paulo State, with increasing urban detection near highways, such as Marechal Rondon, which may act as ecological corridors for vector dispersion^11^ ^,^ ^12^. 

Roadkill monitoring offers insight into local biodiversity and parasite ecology near highways^13^ ^,^ ^14^. This study used the polymerase chain reaction (PCR), a sensitive molecular tool^14^ ^-^ ^16^, to detect *Leishmania* spp. DNA from wild mammals collected along highways in São Paulo State can help elucidate the transmission dynamics in anthropized landscapes.

## METHODS

### Ethics Statement

The study was approved by the Animal Use Ethics Committee (CEUA) of the Faculty of Veterinary Medicine and Zootechnics at the University of São Paulo (CEUAx No. 9327060121) and by the System of Authorization and Information on Biodiversity of the Chico Mendes Institute for Biodiversity Conservation (license number: 77344-1).

### Animals and Study Area

**FIGURE 1:**
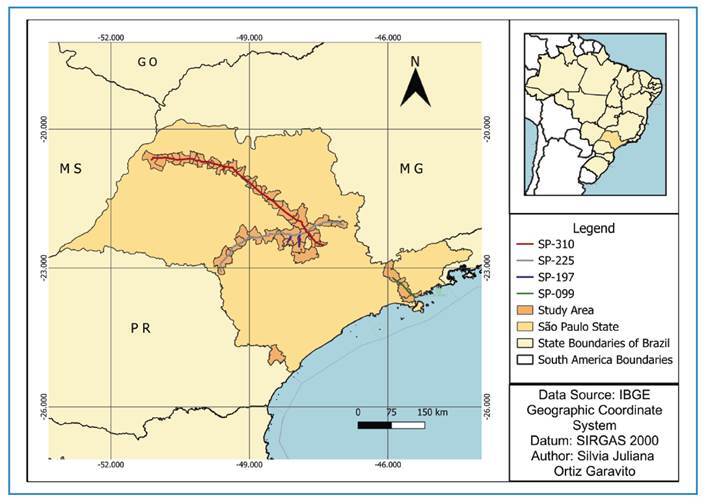
Geographic location of the study areas for road-killed wild mammals along highways in the State of São Paulo. Sections: SP-310: Washington Luiz Highway; SP-225: Eng. Paulo Nilo Romano Highway; SP-197: Doutor Américo Piva Highway; Tamoios Highway, SP-099.

Between 2021 and 2022, 40 roadkilled wild animals were collected along highways in two regions of São Paulo State, Brazil. São Paulo State is situated in the southeastern region of Brazil and covers an area of 248,219.485 km^2^ ^17^. Its geography encompasses two primary biomes: the Atlantic Forest and Cerrado^18^. Our study focused on the municipalities located along highways SP-099, SP-310, SP-225, and SP-197 (Figure 1). 

The first region, which includes fragmented sections of both the Atlantic Forest and Cerrado biomes, covers the northern and midwestern areas and contains highways managed by the Eixo SP that intersect the Cerrado landscape. The first region comprises the Washington Luís Highway (SP-310), which connects Cordeirópolis and Mirassol; the João Ribeiro de Barros/Engenheiro Paulo Nilo Romano Highway (SP-225), which connects cities between Bauru and Itirapina; and the Doutor Américo Piva Highway (SP-197), which runs from Brotas to Torrinha. The second region includes fragmented Atlantic Forest areas along the Serra do Mar and connects São José dos Campos and Caraguatatuba via the Tamoios Highway (SP-099), managed by the Tamoios Concessionaire. Tissue samples from the two species were donated by the Laboratory of Comparative Pathology of the Department of Pathology at the Faculty of Veterinary Medicine and Animal Science, University of São Paulo (USP). These samples were collected along the Régis Bittencourt Highway (BR-116), km 542, in Barra do Turvo, on the roadside in the direction of Curitiba. Wild animals that had been killed by vehicles were collected by concessionaire staff during their 24-hour routine inspections. The bodies were placed in biological material bags, labeled, and stored at −20 °C at each highway's Operational Control Center. The specimens were then transported to the Animal Pathology Service at FMVZ-USP and maintained at 4 °C until necropsy, which was conducted 10-12 h after arrival. During necropsy, the species and age of each animal were confirmed based on the *Mammals of South America* (Volumes 1-3)^19^, and organ samples were collected for molecular analysis.

### Molecular Diagnosis

DNA was extracted from skin tissue samples of the ear pinna using a QIAamp DNA Mini Kit (Qiagen, USA). Briefly, tissue fragments were ground and lysed using a lysis buffer, proteinase K, and ethanol. The material was applied to silica columns and washed with buffers, and DNA was obtained using a kit elution buffer. DNA purity and concentration were determined by spectrophotometry, and the DNA was stored at −20 °C until use. All samples were amplified with mammalian beta-actin primers to ensure DNA quality^20^. For *Leishmania* spp. detection, PCR was performed using the primers Leish-1 (5´-AACTTTTCTGGTCCTCCGGGTAG-3´) and Leish-2 (5´-ACCCCCAGTTTCCCGCC-3´), which amplify a 120-bp fragment of the kinetoplast DNA minicircles (kDNA) from *Leishmania* spp.^21^. The reaction was performed in a final volume of 20 µL containing 10 µL of GoTaq® Green Master Mix 2× (Promega, USA), 0.5 µL of each primer at a concentration of 10 µM (250 nM final concentration in the reaction tube), 5 µL of sterile deionized water, and 4 µL of total DNA from the sample. After an initial denaturation at 95 °C for 5 min, amplification was conducted for 35 cycles under the following program: DNA double-strand denaturation at 95 °C for 15 s, primer annealing at 60 °C for 20 s, and complementary strand extension at 72 °C for 60 s. The final extension was performed at 72 °C for 10 min, after 35 cycles. The PCR products were analyzed by electrophoresis on a 2% (w/v) agarose gel stained with GelRed® (Biotium, USA) for 1.5 h at 100V/100A. The gel was photographed using a photodocumentation system. 

Subsequently, the positive kDNA cases were subjected to a new PCR targeting a 234-bp fragment of the hsp70 gene, and further digested with the *Hae* III enzyme (PCR-restriction fragment length polymorphism [RFLP]) for species identification^22^. PCR-Hsp70 was performed with GoTaq® Green Master Mix (Promega, USA), primers, and sample DNA, according to Graça et al.^22^. After amplification, the products were analyzed by electrophoresis on a 2% agarose gel. For PCR-RFLP, the amplified DNA was incubated with the *Hae* III enzyme and analyzed on a 4% agarose gel. All the reactions included negative and positive controls. 

### Georeferencing

The locations of the cadavers were determined using SIRGAS, a global positioning system device. Coordinates were plotted on digital maps created using the QGIS software, version 3.36.2.

### Statistical Analysis

Descriptive analysis was performed to determine absolute and relative frequencies. The mean distances from the starting points of the highways for the positive, negative, and inconclusive cases were examined to determine differences. Data normality was verified using the Kolmogorov-Smirnov normality test, followed by a one-way analysis of variance (ANOVA) with a significance level of 5% for R^23^.

## RESULTS

In the present study, 14 different species of wild mammals were identified from 40 specimens collected along highways in São Paulo. Carnivora was the most common order, accounting for 72.5% (29/40) of the animals. Pilosa accounted for 20% (n=8/40), whereas rodents and primates accounted for 5% (n=2/40) and 2.5% (n=1/40), respectively. Among the specimens, 52.5% (n=21) were males, and *Tamandua tetradactyla* was the most common species (12.5%; n=5). Females accounted for 47.5% (n=19), with *Leopardus pardalis* being the most common (7.5%; n=3). Of the 40 animals, 75% (n=30) were adults, and 25% (n=10) were juveniles.

The largest number of specimens were collected along the Tamoios Highway (SP-099), accounting for 70% (n=28/40) of the animals analyzed. The Washington Luís Highway (SP-310) contributed 17.5% (n=7/40), followed by the Engenheiro Paulo Nilo Romano Highway (SP-225) at 5% (n=2/40) and the Doutor Américo Piva Highway (SP-197) at 2.5% (n=1/40). The Régis Bittencourt Highway (SP-230) (BR-116) contributed 5% (n=2/40) of all the collected animals.

### Molecular Detection of *Leishmania* spp. DNA in Wild Animals

**FIGURE 2:**
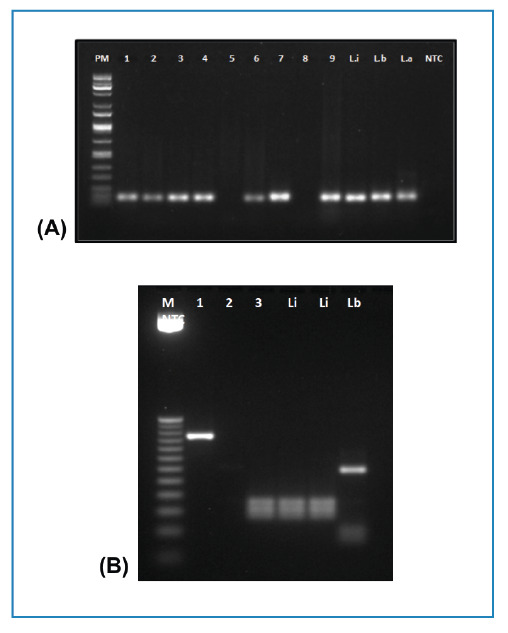
(A) Polymerase chain reaction for *Leishmania* spp in roadkill wild fauna. Lane M: 100 bp DNA ladder. Lanes 1 to 9: representative results of DNA amplification of ear skin samples. Positive controls (120 bp): Li- *Leishmania (L.) infantum chagasi* (MHOM/BR/1972/BH-46); Lb- *Leishmania (V.) braziliensis*, (MHOM/BR/1995/M15280, 26), La- *Leishmania (L.) amazonensis* (MHOM/BR/73/M2269), NTC: No Template Control. **(B)** PCR-RFLP Hsp70 for *Leishmania* identification. Digestion with *Hae* III. Lane M: 50 bp DNA ladder. Lane 1: *Procyon cancrivorus*, Lane 2: *Leopardus pardalis*, Lane 3: *Chrysocyon brachyurus*. Positive controls: Li- *Leishmania (Leishmania) infantum chagasi* (MHOM/BR/1972/BH-46); Li-*Leishmania (Leishmania) infantum* (MHOM/BR/1974/PP75) Lb- *Leishmania (Viannia) braziliensis*, (MHOM/BR/1995/M15280. (MHOM/BR/73/M2269). NTC, no template control.

**FIGURE 3:**
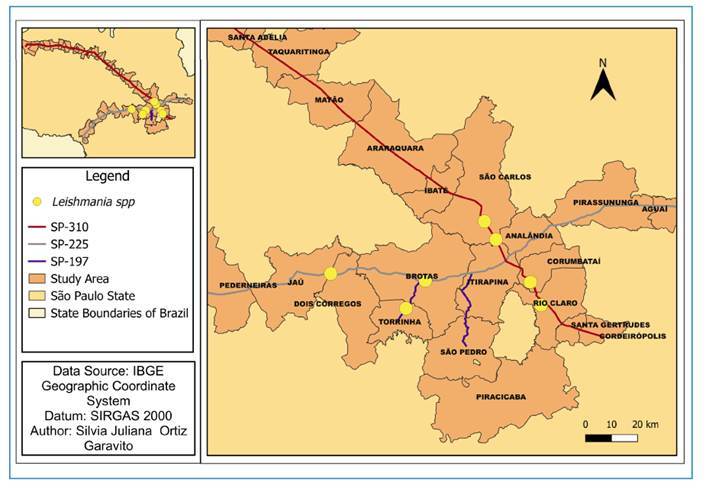
Analysis area 1: Eixo SP Highway Concessionaire - Sections: SP-310: Washington Luiz Highway; SP-225: Eng. Paulo Nilo Romano Highway; SP-197: Doutor Américo Piva Highway. Geographic location of species testing positive for *Leishmania* spp. (yellow points).

Parasite DNA was detected in 29 of the 40 skin samples analyzed by PCR using Leish-1/Leish-2 primers (Figure 2). PCR targeting Hsp70 was then performed on the 29 positive samples, which revealed strong bands in two animals, a racoon (*Procyon cancrivorus*) and a maned wolf (*Chrysocyon brachyurus*), and a weak band in an ocelot (*L. pardalis*). Restriction profiling identified *L. i. chagasi* as the species infecting *C. brachyurus*, a maned wolf, and *Leishmania amazonensis* as the species infecting *P. cancrivorus*, a raccoon. The amplicon of the *L. pardalis* did not generate a restriction profile after *Hae* III digestion (Figure 3, Table 1). Considering the high proportion of kDNA-positive animals, we subjected our primers to Primer-BLAST analysis to assess potential nonspecific primer binding with DNA sequences of other protozoan and metazoan parasites known to circulate among the studied wild fauna (Supplementary Table S1). Primer specificity was confirmed with no alignment with non-*Leishmania* agents.

### Geographic Distribution of Animals Infected with *Leishmania* spp.

**FIGURE 4:**
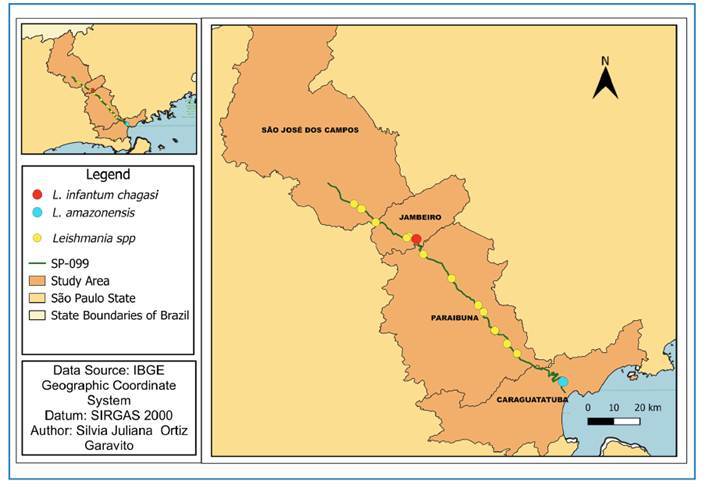
Analysis area 2: Tamoios Highway: SP-099. Geographic location of species testing positive for *Leishmania infantum chagasi* (red points), *Leishmania amazonensis* (blue points), and *Leishmania* spp. (yellow points).

In the present study, *Leishmania* spp. DNA was detected through molecular diagnosis in seven wild mammals killed by vehicle collisions in the first study region, which included highways SP-310 (Corumbataí, São Carlos and Río Claro), SP-225 (Itirapina and Dois Córregos), and SP-197 (Torrinha and Brotas) (Table 1, Figure 3). In the second study region, along SP-099, (Caraguatatuba, Jambeiro, Paraibuna, and São José dos Campos) to *Leishmania* DNA was detected in 23 wild mammals of various species. (Table 1, Figure 4). *L. i. chagasi* DNA was detected in a maned wolf *(C. brachyurus*), and *L. amazonensis* DNA was detected in a raccoon (*P. cancrivorus*), both of which were found in the SP-099 section in Paraibuna and in the Serra do Mar region, Caraguatatuba (Figure 4).

**TABLE 1:** Molecular results for detection of *Leishmania* spp. and species identification in ear pinna skin fragments from road-killed wild mammals on highways in the State of São Paulo, Brazil.

Number of Mammals	Road	Species	PCR Leish 1/2	PCR Hsp70	**RFLP- Hsp70**
					Species identification
1	SP-099	*Procyon cancrivorus*	Positive	Positive	*L. (L.) amazonensis*
4	SP-099	*Tamandua tetradactyla*	Negative	Negative	*-*
5	SP-099	*Nasua nasua*	Positive	Negative	*-*
6	SP-099	*Lontra longicaudis*	Positive	Negative	*-*
7	SP-099	*Tamandua tetradactyla*	Positive	Negative	*-*
8	SP-310	*Chrysocyon brachyurus*	Positive	Negative	*-*
9	SP-099	*Tamandua tetradactyla*	Negative	Negative	*-*
10	SP-099	*Cerdocyon thous*	Positive	Negative	*-*
11	SP-310	*Leopardus pardalis*	Positive	Positive	*-*
12	SP-099	*Procyon cancrivorus*	Positive	Negative	*-*
13	SP-099	*Callithrix aurita*	Positive	Negative	*-*
14	SP-099	*Chrysocyon brachyurus*	Positive	Positive	*L. (L.) infantum chagasi*
15	SP-225	*Puma concolor*	Positive	Negative	*-*
16	SP-310	*Tamandua tetradactyla*	Negative	Negative	*-*
17	SP-099	*Tamandua tetradactyla*	Positive	Negative	*-*
18	SP-225	*Puma concolor*	Positive	Negative	*-*
19	SP-099	*Bradypus variegatus*	Positive	Negative	*-*
20	SP-099	*Leopardus pardalis*	Negative	Negative	*-*
21	SP-099	*Leopardus pardalis*	Positive	Negative	*-*
22	SP-099	*Cerdocyon thous*	Positive	Negative	*-*
23	SP-099	*Cerdocyon thous*	Negative	Negative	*-*
24	SP-099	*Nasua nasua*	Positive	Negative	*-*
25	SP-099	*Nasua nasua*	Positive	Negative	*-*
26	SP-099	*Chrysocyon brachyurus*	Negative	Negative	*-*
27	BR-116	*Lontra longicaudis*	Positive	Negative	*-*
28	BR-116	*Puma concolor*	Positive	Negative	*-*
29	SP-099	*Nasua nasua*	Positive	Negative	*-*
30	SP-099	*Leopardus pardalis*	Positive	Negative	*-*
31	SP-099	*Nasua nasua*	Negative	Negative	*-*
32	SP-099	*Herpailurus yagouaroundi*	Negative	Negative	*-*
33	SP-099	*Cerdocyon thous*	Positive	Negative	*-*
34	SP-197	*Leopardus tigrinus*	Positive	Negative	*-*
35	SP-099	*Galictis cuja*	Positive	Negative	*-*
36	SP-310	*Puma concolor*	Positive	Negative	*-*
37	SP-099	*Coendou spinosus*	Negative	Negative	*-*
38	SP-310	*Tamandua tetradactyla*	Positive	Negative	*-*
39	SP-099	*Leopardus pardalis*	Negative	Negative	*-*
40	SP-099	*Tamandua tetradactyla*	Negative	Negative	*-*
TOTAL=40			Positive=29	Positive= 3	Identification=2

PCR: polymerase chain reaction; Leish-1/ Leish-2: primers; Hsp70: heat shock protein 70; RFLP: restriction fragment length polymorphism.

Statistical analysis along the Tamoios Highway (SP-099) evaluated whether *Leishmania*-positive roadkill was spatially associated with distance from São José.

The distances from São José dos Campos were normally distributed among the positive (n = 20, P = 0.70), negative (n = 6, P = 0.99), and inconclusive (n = 4, P = 0.09) groups. Comparison of the positive (mean = 38.3 km), negative (mean = 51.0 km), and inconclusive (mean = 58.2 km) mean distances revealed no statistically significant differences. No evident spatial pattern was associated with the molecular outcomes within the analyzed area. 

## DISCUSSION

The One Health approach highlights the ecological complexity of leishmaniasis, which involves multiple mammalian hosts across seven orders in the Americas^2^ ^,^ ^24^. Although numerous species show evidence of natural infection, only a limited subset satisfies the World Health Organization-defined reservoir competence, highlighting the need for further studies to elucidate their roles in parasite maintenance and transmission^25^. In the present study, *Leishmania* spp., including *L. infantum chagasi* and *L. amazonensis*, were detected by molecular methods in the ear skin of the sampled animals, even in the absence of visible lesions^26^. The skin is a reliable matrix for PCR-based diagnosis in both symptomatic and asymptomatic hosts^27^ ^,^ ^28^ and is recognized as a primary parasitic reservoir in certain mammalian species^29^. Persistent dermal parasitism, irrespective of clinical status, supports its diagnostic utility and epidemiological relevance^30^ ^-^ ^32^. *Leishmania infantum* DNA detection in the ear skin is comparable to or exceeds that in the bone marrow and spleen of asymptomatic canines^29^ ^,^ ^33^.

In the present study, *Leishmania* spp. DNA was initially screened using PCR targeting the kDNA, a highly sensitive marker owing to its elevated copy number within the parasite genome^21^ ^,^ ^34^ ^,^ ^35^. Field-collected specimens such as roadkilled animals may contain partially degraded tissues. Owing to the relative stability of DNA and high sensitivity of PCR, parasite DNA can still be detected after exposure to adverse conditions^36^ ^,^ ^37^, making kDNA-PCR particularly suitable for degraded or low-quality samples, such as those obtained from wildlife carcasses. Regarding specificity, kDNA-PCR has been validated in previous studies, which have demonstrated no amplification of DNA from non-*Leishmania* parasites or other microorganisms commonly present in endemic areas^38^ ^,^ ^39^. To further support the specificity of kDNA-PCR, primers Leish-1 and Leish-2 were subjected to primer-BLAST analysis, which revealed no alignment with DNA from metazoan or protozoan parasites that commonly infect wild fauna collected in this study. Although we did not sequence the amplicons to independently confirm their specificity, these findings support the use of kDNA-PCR as a sensitive and reliable screening tool for detecting *Leishmania* DNA in challenging wildlife samples.

To examine the specificity of another molecular reaction and particularly to enable species-level identification, positive kDNA samples were further analyzed by PCR-RFLP targeting the *hsp70* gene^40^ ^-^ ^42^. Although this gene is considerably less sensitive because it is present in a single copy, it provides high accuracy in distinguishing *Leishmania* species^22^ ^,^ ^41^ ^,^ ^42^. Our findings support those of previous studies that identified *Leishmania* spp. DNA from maned wolves (*C. brachyurus*) even without visible skin lesions^41^. Similarly, *Leishmania* spp. have been reported in *Cerdocyon thous* using PCR-kDNA, although PCR-Hsp70 failed to confirm the infection^43^. This could be attributable to the significantly higher number of kDNA copies in the genome compared to other targets. By contrast, nuclear genes with limited copy numbers such as Hsp70 exhibit lower sensitivity when the parasitic DNA is scarce^44^. A similar scenario may have occurred in the present study, as only 3 of 29 kDNA-positive animals presented detectable Hsp70 bands. The species was identified in two cases after enzymatic digestion with *Hae* III. Of the 29 kDNA-positive samples, only two yielded interpretable results with *hsp70*-RFLP: one maned wolf (*C. brachyurus*) infected with *L. i. chagasi* and one raccoon (*P. cancrivorus*) infected with *L. amazonensis*. The low number of *Leishmania* species identified could likely reflect the reduced sensitivity of the hsp70 marker and tissue degradation in roadkilled specimens. 

*L. amazonensis* occurs in a diverse range of wild mammals in South America^2^. However, *Leishmania* species being detected in procyonids has not been previously recorded; thus, to the best of our knowledge, our study is the first to report the molecular detection of *L. amazonensis* DNA in raccoons (*P. cancrivorus*) in this region.

This study reports the first detection of *L. i. chagasi* in a maned wolf in the Paraibuna region*. L. i. chagasi* has been previously reported in carnivores, including *Cerdocyon thous* ^44^ ^,^ ^45^ and *C. brachyurus* ^46^ ^,^ ^47^. Previous studies on the potential role of carnivores, such as *C. thous* as reservoirs for *L. i chagasi* ^48^ ^,^ ^49^ and hosts for *L. amazonensis* ^50^ ^,^ ^51^, have reported that reservoir competence depends on infection prevalence and ability to transmit *Leishmania* to vectors. Additionally, four *Nasua nasua* individuals (4/40) tested positive for *Leishmania* spp. DNA, which aligned with the previous findings from Mato Grosso do Sul^52^. Similarly, *Leishmania* spp. DNA was detected in *Puma concolor* (n=4/40) and *L. pardalis* (n=3/40), confirming the documented infections in Brazilian carnivores^53^ ^,^ ^54^.

In this study, *Leishmania* spp. DNA was detected in *Lontra longicaudis* (n=2/40), corroborating previous findings of infection in captive otters of the Brasília Zoo *Leishmania* spp. DNA was detected in two *L. longicaudis* individuals (n=2/40)^55^. Furthermore, *Leishmania* spp. were detected only *in Galictis cuja* (n=1/40) in our study. This is consistent with prior serological detection of this species in Botucatu, São Paulo^56^.

*Leishmania* spp. were detected in *Tamandua tetradactyla* (Pilosa) in São Paulo (2014), echoing the positivity for *Leishmania* spp. identified in three collared anteaters (n=3/40) in our study. *Bradypus variegatus* (n=1/40) was the only sloth species identified in this study, although *L. (Viannia) shawi* has previously been reported in *Bradypus tridactylus* from Pará^31^. *L. hertigi* was identified in *Coendou prehensilis*, which is in the Rodentia order, in Piauí and Ceará^57^ ^,^ ^58^. In the present study, one porcupine (*Coendou spinosus*) tested positive for *Leishmania* spp. Additionally, the present study detected *Leishmania* spp. DNA from several wild mammalian species for which no previous records have been found. *Leopardus tigrinus* (n=1/40) and *Bradypus variegatus* (n=1/40) tested positive for *Leishmania* spp. DNA for the first time in this context.

In this study, *Leishmania* spp. DNA was detected in wild mammals along the SP-310 highway, encompassing the municipalities (Cordeirópolis, Mirassol, Rio Claro, São Carlos, Araraquara, Matão, Catanduva, and São José do Rio Preto) where *Lu. longipalpis*, the primary vector of *L. infantum*, has been reported^59^. The vector’s progressive expansion into São Paulo’s interior, especially in urban areas near highways such as Marechal Rondon correlates with the spread of AVL^60^. Entomological surveys have confirmed *Lu. longipalpis* in periurban zones of Rio Claro and Corumbataí between 2001 and 2004^61^. The municipalities of Cordeirópolis, São Carlos, and Araraquara were classified as silent but receptive areas, whereas Mirassol and São José do Rio Preto exhibited human and canine transmission^62^. Additionally, *Leishmania* spp. DNA was also detected along the SP-225 and SP-197 highways. Previous reports of sustained vector presence in municipalities, such as Cordeirópolis, Bauru, and Botucatu, have suggested the role of ecological corridors and highway expansion^63^ ^,^ ^64^. 

Besides *Lu. Longipalpis*, secondary vectors such as *Pintomyia fischeri* and *Mi. migonei* have been recorded in the peri-urban areas of Bauru, Itirapina, and Brotas. *P. fischeri* showed natural infection (~4.8%) and infectivity comparable to those of *Lu. Longipalpis* ^65^ *,* whereas *M. migonei* supports *L. infantum* development experimentally^66^_._ *Nyssomyia whitmani* is an established vector of *L. braziliensis* ^67^. Among surveyed sites, Analândia and Itirapina are silent but receptive, Jaú has human cases, and Bauru remains a high-transmission priority area^62^. Similarly, in this study, *Leishmania* spp. DNA was detected in *P. concolor* and *L. tigrinus* in the municipalities of Brotas, Dois Córregos, and Torrinha and in *L. pardalis*, *C. brachyurus*, and *Tamandua tetradactyla* in São Carlos, Itirapina, and Rio Claro. Previous studies have demonstrated the presence of *Leishmania* spp. in various wild mammals in the study region. Bauru is one of the most affected cities in the state with 381 confirmed cases reported between 2004 and 2012^61^. The detection of *Leishmania* DNA in *Didelphis* spp. suggests their potential involvement in urban transmission^68^. Surveys near Campinas confirmed *L. infantum* infection in multiple hosts and vectors, indicating an active sylvatic transmission cycle in areas with human and canine infections since the 1990s^69^.

In the SP-099 region of northern coastal São Paulo, *Leishmania Infantum chagasi* DNA was detected in a maned wolf (*C. brachyurus*) in Paraibuna, and *L. amazonensis* DNA in a raccoon (*P. cancrivorus*) in peri-urban Caraguatatuba, areas where vectors *Ny. intermedia*, *Ny. neivai* and *P. fischeri* are reported^61^ ^,^ ^62^ ^,^ ^70^. In Guarujá, human and canine VL cases were associated with *Ny. intermedia* dominance^71^. Caraguatatuba reported 689 American cutaneous leishmaniasis (ACL) cases from 1993 to 2005, and 146 ATL cases between 2007 and 2020, with *Ny. intermedia* as the main vector^70^ ^,^ ^72^. Co-circulation of *L. braziliensis* and *L. amazonensis* has also been observed in small sympatric mammals^70^ ^,^ ^73^. These findings demonstrated the presence of *Leishmania* spp. DNA from wild mammals in the analyzed regions of São Paulo. Our findings align with those of previous reports of *Leishmania* exposure in free-ranging wild mammals^14^ ^,^ ^15^, indicating parasite circulation in the natural environment. The absence of case clusters suggests endemicity with low incidence, reinforcing the need for passive surveillance, as the disease tends to persist and expand once established^74^. Molecular tools detect *Leishmania* DNA in several wild mammal species, providing new data from understudied areas of São Paulo. Although the use of roadkill samples allowed non-invasive access to rare or elusive species, it also imposed limitations, such as the inability to assess clinical status, parasite load, or vector interactions, the condition of the carcasses and potential post-mortem degradation, which may have influenced DNA quality and detection sensitivity. The opportunistic nature of the sample collection could also introduce spatial and temporal biases. Nevertheless, roadkill sampling could provide non-invasive access to elusive species, confirming local *Leishmania* circulation in sylvatic areas and highlighting the value of passive and integrative surveillance for assessing exposure in wild mammals^14^ ^,^ ^15^. 

## CONCLUSIONS

Molecular screening of ear skin tissue from roadkilled wild mammals in different regions of the State of São Paulo enabled the detection of *Leishmania* spp. DNA from 29 samples of different wild mammalian species Species-level identification using hsp70-RFLP confirmed the presence of *L. i. chagasi* in a maned wolf (*C. brachyurus*), representing the first record of this parasite species in the Paraibuna region. *L. amazonensis* was identified in a raccoon *(P. cancrivorus)* from Caraguatatuba, located in the Serra do Mar region on the northern coast of the state, suggesting that these animals are exposed to different *Leishmania* species across distinct ecological settings.

The use of roadkill specimens may offer a valuable non-invasive tool for pathogen surveillance in elusive wildlife species, supporting the One Health approach by enhancing the understanding of parasite dynamics in data-deficient regions.

## Data Availability

All data supporting the findings of this study are included in the article and are available without restriction.
